# HMGB3 promotes growth and migration in colorectal cancer by regulating WNT/β-catenin pathway

**DOI:** 10.1371/journal.pone.0179741

**Published:** 2017-07-05

**Authors:** Zheying Zhang, Yaya Chang, Jianming Zhang, Yanxia Lu, Lin Zheng, Yuhan Hu, Fan Zhang, Xiaomin Li, Wenjuan Zhang, Xuenong Li

**Affiliations:** 1 Department of Pathology, School of Basic Medical Sciences, Southern Medical University, Guangzhou, China; 2 Department of Pathology, Xinxiang Medical University, Xinxiang, Henan, China; 3 ShijingShan Teaching Hospital of Capital Medical University, Beijing, China; 4 Department of General Surgery, Nanfang Hospital, Southern Medical University, Guangzhou, China; Beijing Cancer Hospital, CHINA

## Abstract

Colorectal cancer (CRC) is the third leading cause of cancer-related deaths and a major health problem. High mobility group box 3 (HMGB3), a member of the high-mobility group box (HMGB) family, was reported to be over-expressed in gastric carcinoma and bladder cancer. However, the function of HMGB3 in CRC remains unclear. Here, we found that HMGB3 was up-regulated in CRC at both mRNA and protein levels. qRT-PCR results showed that high expression of HMGB3 had positive correlation with serosal invasion, lymph metastasis, and tumor–node–metastasis (TNM) stage in CRC patient. Functional experiments showed that HMGB3 can promote CRC cells proliferation and migration in vitro. Moreover, we found HMGB3 can active WNT/β-catenin pathway to increase the expression level of c-Myc and MMP7. These results may be the reason for HMGB3 oncogene role in CRC. In summary, our data indicated that HMGB3 may serve as an oncoprotein and could be used as a potential prognostic marker in CRC.

## Introduction

Colorectal cancer (CRC) is a common malignant tumor in the digestive system [1]. In recent years, the incidence of CRC is increasing year by year. Approximately 1.2 million patients worldwide are diagnosed with CRC each year, and more than 600 thousand patients died directly or indirectly of CRC [2–4]. Early signs of CRC are not obvious, symptoms often appear late and prone to metastasis, then the prognosis is poor [5]. This is the main reason for the high mortality rate. Therefore, it is imperative to identify diagnostic factor for CRC in early stage and investigate their functions in CRC.

High mobility group box 3 (HMGB3) is a member of the high-mobility group box (HMGB) family, which including HMGB1, HMGB2, HMGB3, HMGB4 [6]. The HMG-Box subfamily plays an important role in DNA replication, transcription, recombination and repair [7–9]. HMGB3 was 80% identity with HMGB1 and HMGB2 [6], suggests similar functions at the molecular level. HMGB1 and HMGB2 have been reported played an important role in cancer [10–13]. Furthermore, previous studies have shown that HMGB3 participated in some types of cancers progression, such as urinary bladder cancer, esophageal squamous cell cancer, gastric cancer, non-small cell lung cancer, breast cancer [14–18]. However, the expression and role of HMGB3 in human CRC remain unclear. Therefore, in this study we will detect HMGB3 expression level in CRC, determine the relationship between HMGB3 expression and clinical pathological parameter, analyze the function and molecular mechanism of HMGB3 in growth and migration of CRC.

## Materials and methods

### Clinical specimens and cell lines

Human colorectal cancer tissues and paired normal colorectal mucosa tissues were collected from Nanfang Hospital, Southern Medical University (Guangzhou, China), and written informed consent was obtained from all patients or their relatives. All the human work was approved by the Medical Ethics Committee of Nanfang Hospital, Southern Medical University. The tissue specimens were frozen in liquid nitrogen and stored at -80°C. The CRC cell lines used in this research were obtained from ATCC and cultured in RPMI 1640 (Hyclone) supplemented with 10% fetal bovine serum (FBS) (Gibco) at 37°C with 5% CO2.

### RNA extraction and qRT-PCR

TRIzol reagent (Takara) was used to extract tissues and cells RNA according to the manufacturer’s instructions. Reverse Transcription Kit (Takara) was used to transcribe RNA to cDNA. Quantitative real-time PCR (qRT-PCR) analyses were performed with SYBR GreenⅠ(Takara) in triplicates. qRT-PCR was used to analyse the expression level of HMGB3 in CRC. HMGB3 expression was normalized to GAPDH and the results were presented as the fold-change in tumor tissues relative to the matched adjacent normal tissues. Formula Folds = 2-^ΔΔCt^ was used to calculate relative expression levels of HMGB3 in tissues. ΔCt values were used to compare expression level of HMGB3 in tumor and control group. ΔCt = Ct_HMGB3_ –Ct_GAPDH,_ ΔΔCt = ΔCt_Tumor_ –ΔCt_Normal._. The HMGB3 primers are listed as follows. The forward primer 5′- ATTCGGAATTCCGTATCTGGCCTTTTGAC-3′ and the reverse primer 5′- CGGTTACTCGGCTTACGCTTGGACTG -3′; GAPDH was used as an internal control. The primers for GAPDH were 5′-GACTCATGACCACAGTCCATGC-3′ and 5′- AGAGGCAGGGATGATGTTCTG -3′.

### Immunohistochemistry (IHC)

The primary tumors were immunostained for HMGB3 as previously described [19]. The HMGB3 index was calculated as that the number of HMGB3 positive cells divided by the number of total cells ×100% (magnification, ×200).

### Construction of plasmids and transfection

Lentiviral constructs repressing HMGB3 was purchased from Genechem (Shanghai, China) and were used to establish cells lines constitutively repressing HMGB3. The nucleotide sequences of siRNA for HMGB3 were listed as following. Sense 5’-GACUAUAAGUCGAAAGGAATT-3’, antisense 5’-UUCCUUUCGACUUAUAGUCTT-3’. The full length of HMGB3 was amplified by PCR using cDNA from non-tumour colorectal mucosal tissues, then cloned into pcDNA3.0 vectors. Cells were transfected with plasmids using Lipofectamine 2000 (Invitrogen).

### Cell proliferation, colony formation and transwell assays

The proliferation, plate colony formation, invasion, and migration of transfected CRC cells were examined as previously described [20].

### Flow cytometry

The cells were harvested after transfecting 24 later. Fluorescein isothiocyanate (FITC) Annexin V and propidium iodide were used to stain cells according to the FITC Annexin V Apoptosis Detection Kit (BD Biosciences) protocol. Then, a flow cytometric analysis was used to determine the cells number in G0–G1, S, and G2–M phases.

### Western blot assay (WB)

Western blot was performed as previously described [21]. Antibodies of HMGB3, β-catenin, c-Myc, MMP7, GAPDH, β-actin was purchased from Abcam. Protein expression level of HMGB3, β-catenin, c-Myc, MMP7 was normalized to GAPDH or β-actin and quantified using Image J software.

### TOP-Flash WNT reporter

The activity of the WNT pathway was examined using a TOP-Flash luciferase reporter. Cells were co-trasfected with 250ng TOP-FLASH or FOP-FLASH and 25ng pRL-SV40 plasmid. Luciferase activity was measured by the Dual-Luciferase Reporter Assay System (Promega). The ratios of TOP/FOP were calculated and used as indicators of WNT signaling activity [22].

### Statistical analyze

Data was analyzed by SPSS 20.0 Statistical software. Quantitative data was presented as the mean ± SD of at least 3 independent experiments. The differences between independent experimental groups were tested by using a two-tailed Student's *t*-test. Relationships between HMGB3 expression and clinicopathologic characteristics were determined by χ^2^ test. Differences were considered significant if *p* < 0.05: *, *p* < 0.05; **, *p* < 0.01; ***, *p* < 0.001.

## Results

### Increasing of HMBG3 correlated with CRC progression

**Fig 1:**
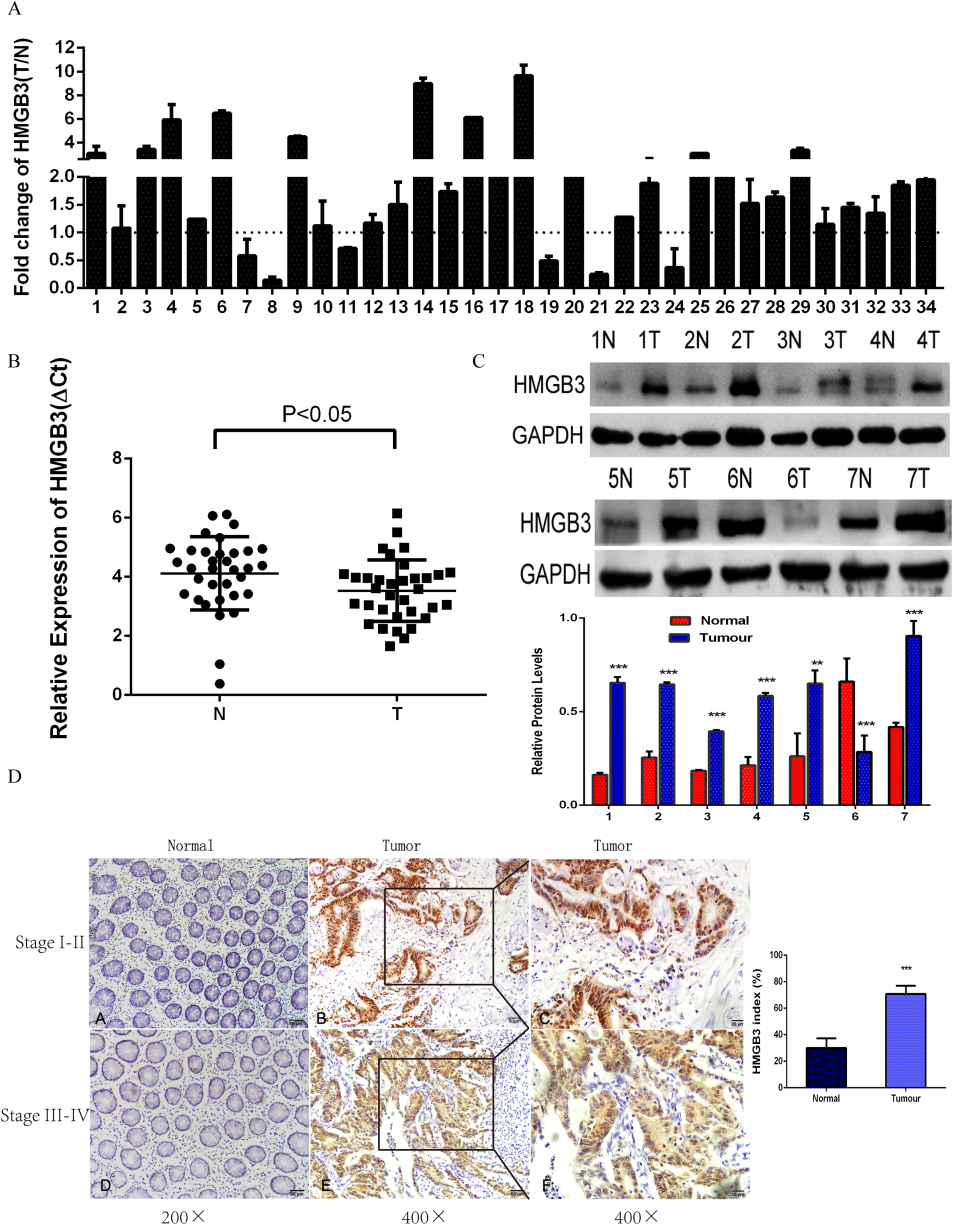
Expression levels of HMGB3 mRNA and protein are increased in CRC tissues. (A) qRT-PCR analysis of HMGB3 expression in 34 paired CRC tissues and adjacent normal tissues. HMGB3 expression level was normalized to GAPDH and the results were presented as the fold-change in tumor tissues relative to the matched adjacent normal tissues. Error bars indicate mean ± standard deviation of 3 independent experiments. (B) The value of ΔCt was used to show the expression level of HMGB3 (ΔCt = Ct_HMGB3_ –Ct_GAPDH_) in the 34 paired human CRC tissues (T) and adjacent normal tissues (N) (*p* < 0.05). The differences between tumor group and normal group were tested by using a Paired-samples *t*-test. (C) Western blot analysis of HMGB3 protein expression in seven pair CRC tissues compared with the normal tissues. The differences between tumor group and normal group were tested by using a Independent-Samples t-test. (D) Representative HMGB3 staining in CRC specimens. Left, normal tissue, Scale bars *=* 50 μm. Middle, CRC tissues, Scale bars = 50μm. Right, CRC tissues, Scale bars *=* 20 μm. The HMGB3 index was calculated as that the number of HMGB3 positive cells divided by the number of total cells ×100% (magnification, ×200). The differences between tumor group and normal group were tested by using a Independent-Samples t-test. Error bars represent the mean ± SD of 5 different fields. ***, *p* < 0.001.

To investigate the role of HMBG3 in CRC tumorigenesis, the expression levels of HMBG3 were determined in 34 paired CRC tissues and adjacent normal counterparts by qRT-PCR. HMGB3 expression was normalized to GAPDH and the results were presented as the fold-change in tumor tissues relative to the matched adjacent normal tissues in Fig 1A. Formula Folds = 2-^ΔΔCt^ was used to calculate relative expression levels of HMGB3 in tissues. ΔCt = Ct_HMGB3_ –Ct_GAPDH,_ ΔΔCt = ΔCt_Tumor_ –ΔCt_Normal._. Paired-samples t test was used to analyse ΔCt values of tumor and control group in Fig 1B. The results revealed HMBG3 expression was increased in 28 of 34 CRC specimens (P < 0.05) (Fig 1A and 1B). We next divided the level of HMBG3 into a high-expression group (*n* = 18) and a low-expression group (*n* = 16) by the median of HMBG3 expression level and examined the relationship between HMBG3 expression levels and the clinicopathological characteristics of the tumor tissue samples. Correlation analysis showed that HMBG3 expression was positively associated with serosal invasion, lymph metastasis, and tumor–node–metastasis (TNM) stage in CRC (Table 1). In addition, western blot assay was used to determine the protein level of HMGB3 in 7 paired CRC tissues. The results showed the protein expression of HMGB3 in CRC tissues was higher compared with paired adjacent normal tissues detected by western blot (P<0.05). Among them, the protein level of HMGB3 of 6N is higher than 6T. Probably because of protein degradation, and may also be due to individual differences in the patient (Fig 1C). We further confirmed HMGB3 protein expression level was up-regulated in 50 pair CRC tissues compared with adjacent normal tissues by using IHC assay (P<0.001) (Fig 1D).

**Table 1:** Clinicopathologic characteristics of HMGB3 expression in CRC patients.

Clinicopathological variables	Expression of HMGB3		P Value
	Low expression	High expression	
N	16	18	
Gender			
Male	11	16	0.327
Female	5	2	
Age^a^			
<52	6	10	0.214
≥52	10	8	
Tumor Size(cm)^b^			
<4	6	7	0.607
≥4	10	11	
Differentiation			
well	6	7	0.607
Moderate or poor	10	11	
Serosal Invasion			
Yes	5	14	0.014
No	11	4	
Lymph Metastasis			
Yes	6	14	0.035
No	10	4	
TNM classification			
Ⅰ-Ⅱ	13	2	0.012
Ⅲ-Ⅳ	3	16	

^a^ Group of age was performed according to median.

^b^ Tumor size was grouped according to median.

The tumor samples were divided into a high-expression group (*n* = 18) and a low-expression group (*n* = 16) by the median of HMBG3 expression level and χ^2^ test was used to examine the relationship between HMBG3 expression levels and the clinicopathological characteristics of the tumor tissue samples.

### HMGB3 promotes CRC cell lines proliferation and migration in vitro

**Fig 2:**
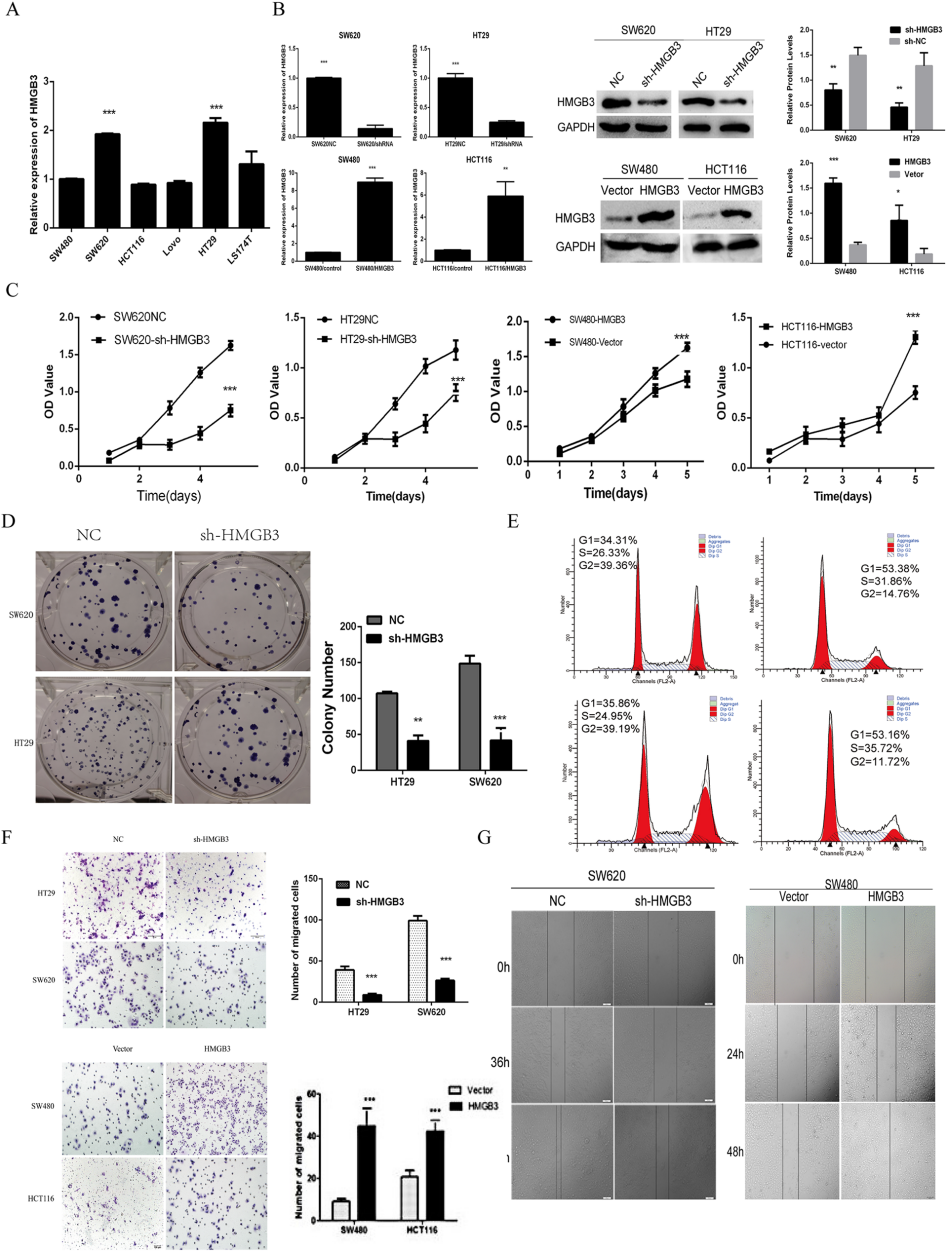
HMGB3 promote CRC cells proliferation and migration in vitro. (A) qRT-PCR analysis of HMGB3 expression in six CRC cell lines. The differences between independent experimental groups were tested by using Independent-Samples t-test. Error bars indicate mean ± SD of 3 independent experiments. ***, *p* < 0.001. (B) qRT-PCR and western blot analyses of HMGB3 mRNA and protein levels after the transfection of the sh-HMGB3 or HMGB3 plasmid. The differences between independent experimental groups were tested by using Independent-Samples t-test. ***, *p* < 0.001. (C) Effects of sh-HMGB3 and oe-HMGB3 on cell proliferation were determined by CCK8 cell proliferation assay. Error bars represent the mean ± SD of 5 independent experiments. The differences between independent experimental groups were tested by using Independent-Samples t-test. ***, *p* < 0.001. (D) The proliferation ability was determined by colony formation assay of the cell SW620 and HT29 after inhibiting the expression of HMGB3. The bar chart represents the colony number. Error bars represent the mean ± SD of 3 independent experiments. The differences between independent experimental groups were tested by using Independent-Samples t-test. **, *p* < 0.01; ***, *p* < 0.001. (E) Cell cycle G1 arrest after knocking down HMGB3 measured by flow cytometry. (F) The migration capacity was determind by transwell assays. The bar chart represents the migration cell numbers. Error bars represent the mean ± SD of 5 different field. The differences between independent experimental groups were tested by using Independent-Samples t-test. ***, *p* < 0.001. (G) Effects of sh-HMGB3 or oe-HMGB3 on cell migration ability were determined by Wound healing assay.

To gain insight into the function of HMGB3 in CRC progression, we generated stable cell lines for HMGB3 over-expression or knockdown. According to the results of HMGB3 expression level in CRC cell lines (Fig 2A), we choose SW480 and HCT116 which have lower expression level to establish stable cell lines over-express HMGB3, SW620 and HT29 which have higher expression level to establish stable cell lines inhibiting HMGB3 expression (Fig 2B). Cell Counting Kit-8 (CCK8) proliferation assays showed that inhibition of HMGB3 expression level decreased cell growth. Inversely, SW480-pcDNA3.0-HMGB3 and HCT116-pcDNA3.0-HMGB3 cells showed higher proliferative capacity compared with that of negative control (NC) cells (Fig 2C). Similarly, colony formation capacity was suppressed after knockdown of HMGB3 (Fig 2D). Furthermore, flow cytometry showed that the down-regulation of HMGB3 in SW620 and HT29 cells significantly increased the proportion of cells in G0/G1 phase, while decreased the proportion of cells in S phase (Fig 2E). Transwell and wound healing assay were used to determine cell motility, the results revealed that repression of HMGB3 could attenuate the migration of SW620 and HT29 cells. Inversely, SW480-pcDNA3.0-HMGB3 and HCT116-pcDNA3.0-HMGB3 cells showed higher migratory ability compared with that of NC cells (Fig 2F and 2G).

### HMGB3 regulates the genes expression of WNT/β-catenin pathway

**Fig 3:**
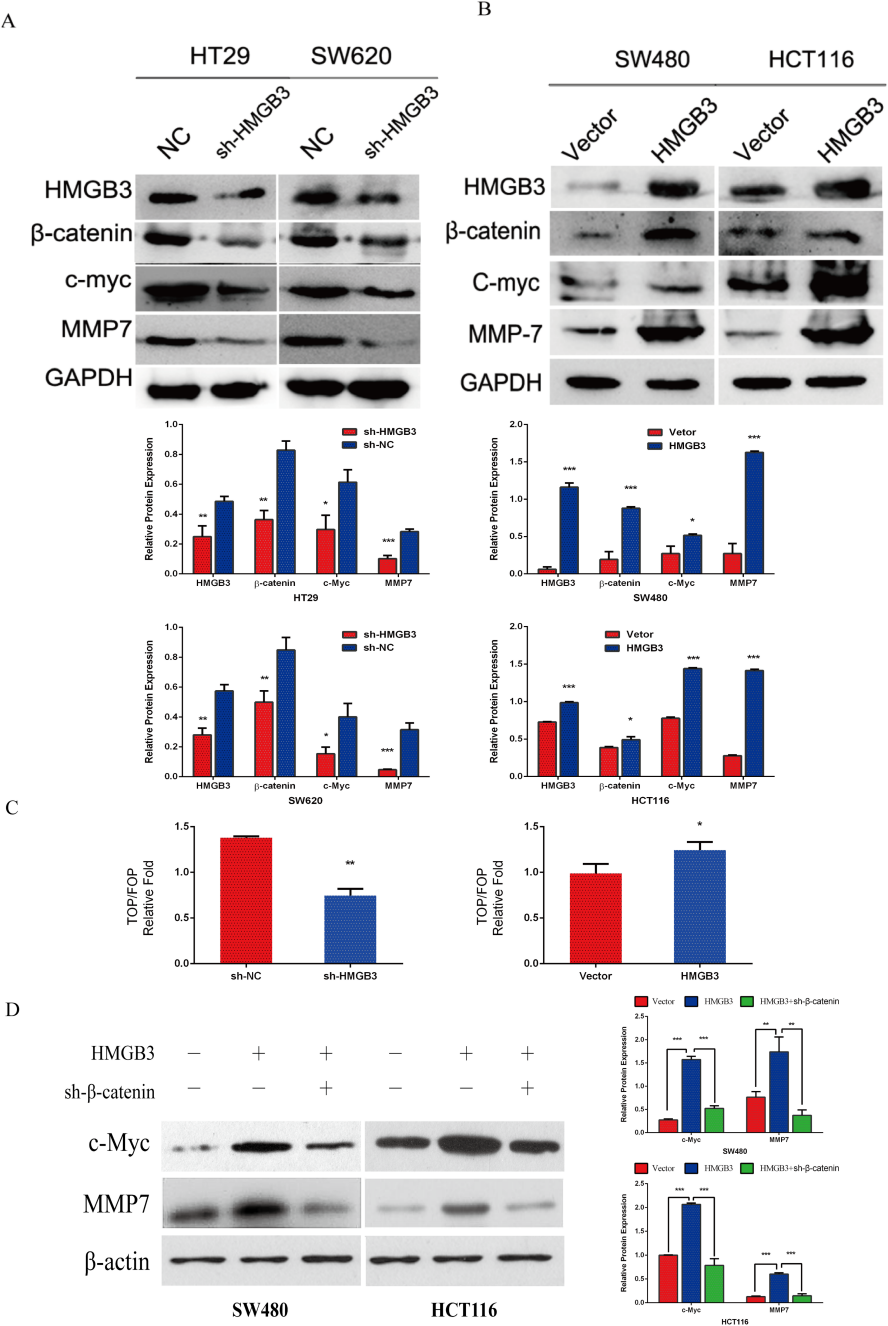
HMGB3 promotes CRC cell growth and migration through WNT/β-catenin pathway. Western blot was performed to detect HMGB3, β-catenin, c-Myc, and MMP7 expression after hmgb3 knocking down (A) or up-regulating (B). Data are mean ± SD for triplicate samples. GAPDH served as the loading control. The differences between independent experimental groups were tested by using Independent-Samples t-test. (C) Cells were transfected with TOP-Flash or control FOP-Flash reporter to determine reporter activities 48h later. The values of TOP-Flash and FOP-Flash were normalized to the value of pRL-SV40, the bar chart represented the ratios of TOP/FOP. Values are mean ± SD for triplicate samples. The differences between independent experimental groups were tested by using Independent-Samples t-test. *, *p* < 0.05; **, *p* < 0.01. (D) The expression of c-Myc, MMP7 and β-actin in SW480 and HCT116 cells were detected by western blot after increasing the expression of HMGB3 or increasing the expression of HMGB3 but inhibited β-catenin expression. β-actin served as the loading control. Error bars represent the mean ± SD of 3 independent experiments. The differences between independent experimental groups were tested by using Independent-Samples t-test. **, *p* < 0.01; ***, *p* < 0.001.

To examine how HMGB3 is playing a role in CRC progression. We determine the protein expression levels of WNT pathway such as β-catenin, c-Myc and MMP7. The result showed the protein level ofβ-catenin, c-Myc and MMP7 was increased after up-regulating the expression of HMGB3 (Fig 3A). On the contrary, the protein level of these genes was decreased after knocking down the expression of HMGB3 (Fig 3B). We hypothesized that HMGB3 could modulate Wnt/β-catenin signaling. To test the hypothesis that HMGB3 plays an important role in activating WNT signaling, we used a TOP-Flash luciferase assay and observed that down-regulation of HMGB3 inhibited WNT/β-catenin pathway activity. On the other hand, over-expression of HMGB3 up-regulated WNT/β-catenin signaling activity (Fig 3C). Furthermore, we found that inhibition of β-catenin abolished the promoting effect of HMGB3 in WNT pathway target genes protein expression level (Fig 3D).

## Discussion

CRC is a major health problem as it constitutes the third leading cause of cancer-related deaths. As other malignant tumors, the mechanism for CRC progression remains unclear. Previous studies have suggested HMGB3 involved in many carcinogenesis and progression. Such as gastric cancer [16,22], leukemia [23], breast cancer [18], and lung cancer [17]. But little is known about the function of HMGB3 expression in CRC. In this study, we investigated HMGB3 expression, function and a simple mechanism in CRC. Compared with the normal group, HMGB3 is pathologically up-regulated in CRC cells and tissues. Moreover, HMGB3 up-regulation in CRC patients is associated with tumor serosal invasion, lymph metastasis, and TNM classification. Our results indicate that over-expression of HMGB3 may be a common incident in CRC and HMGB3 seems to function as an oncogene during CRC cancergenesis and progression.

Additionally, we found that suppression of HMGB3 decreases the ability of CRC cells in growth and migration. Conversely, increasing of HMGB3 promoted CRC cell proliferation and invasion. Therefore, the results showed that HMGB3 exerts oncogene effect to promote cell growth and migration in CRC.

The mechanism of HMGB3 in promoting CRC progression is still uncertain. 90% CRC showed aberrant activity of WNT/β-catenin signaling. Then, we examined the expression of key proteins in WNT pathway. We found HMGB3 could up-regulate β-catenin which is a key gene of WNT pathway and downstream gene c-Myc and MMP7 expression level. We hypothesized that HMGB3 could modulate Wnt/β-catenin signaling. Then, we examined activity of Wnt/β-catenin signaling through over-expression or knockdown of HMGB3. The results showed that HMGB3 could regulate WNT/β-catenin pathway activity. Furthermore, we found that inhibition of β-catenin abolished the promoting effect of HMGB3. Therefore, we speculated that HMGB3 promoted CRC cell growth and migration through WNT/β-catenin pathway.

In summary, our results indicate that HMGB3 is a tumor promotor, when inhibit, may suppress CRC proliferation and migration. Role of HMGB3 in cancergenesis and development of CRC relates with regulating activity of WNT/β-catenin pathway. Our results indicate that HMGB3 may be a promising therapeutic target for CRC.

## References

[1] Herszenyi L, Tulassay Z. Epidemiology of gastrointestinal and liver tumors. Eur Rev Med Pharmacol Sci. 2010; 14: 249–258. 20496531

[2] Emmons KM, Burns White K, Benz EJ. Development of an integrated approach to cancer disparities: one cancer center's experience. Cancer Epidemiol Biomarkers Prev. 2007; 16: 2186–2192. doi: 10.1158/1055-9965.EPI-07-0211 18006905 10.1158/1055-9965.EPI-07-0211

[3] Center MM, Jemal A, Smith RA, Ward E. Worldwide variations in colorectal cancer. CA Cancer J Clin. 2009; 59: 366–378. doi: 10.3322/caac.20038 19897840 10.3322/caac.20038

[4] Bray F, Ren JS, Masuyer E, Ferlay J. Global estimates of cancer prevalence for 27 sites in the adult population in 2008. Int J Cancer. 2013; 132: 1133–1145. doi: 10.1002/ijc.27711 22752881 10.1002/ijc.27711

[5] Calon A, Espinet E, Palomo-Ponce S, Tauriello DV, Iglesias M, Cespedes MV, et al Dependency of colorectal cancer on a TGF-beta-driven program in stromal cells for metastasis initiation. Cancer Cell. 2012; 22: 571–584. doi: 10.1016/j.ccr.2012.08.013 23153532 10.1016/j.ccr.2012.08.013 PMC3512565

[6] Nemeth MJ, Curtis DJ, Kirby MR, Garrett-Beal LJ, Seidel NE, Cline AP, et al Hmgb3: an HMG-box family member expressed in primitive hematopoietic cells that inhibits myeloid and B-cell differentiation. Blood. 2003; 102: 1298–1306. doi: 10.1182/blood-2002-11-3541 12714519 10.1182/blood-2002-11-3541

[7] Agresti A, Bianchi ME. HMGB proteins and gene expression. Curr Opin Genet Dev. 2003; 13: 170–178. 12672494 10.1016/s0959-437x(03)00023-6

[8] Stros M. HMGB proteins: interactions with DNA and chromatin. Biochim Biophys Acta. 2010; 1799: 101–113. doi: 10.1016/j.bbagrm.2009.09.008 20123072 10.1016/j.bbagrm.2009.09.008

[9] Kostova N, Zlateva S, Ugrinova I, Pasheva E. The expression of HMGB1 protein and its receptor RAGE in human malignant tumors. Mol Cell Biochem. 2010; 337: 251–258. doi: 10.1007/s11010-009-0305-0 19876719 10.1007/s11010-009-0305-0

[10] Kang R, Chen R, Zhang Q, Hou W, Wu S, Cao L, et al HMGB1 in health and disease. Mol Aspects Med. 2014; 40: 1–116. doi: 10.1016/j.mam.2014.05.001 25010388 10.1016/j.mam.2014.05.001 PMC4254084

[11] Wang X, Xiang L, Li H, Chen P, Feng Y, Zhang J, et al The Role of HMGB1 Signaling Pathway in the Development and Progression of Hepatocellular Carcinoma: A Review. Int J Mol Sci. 2015; 16: 22527–22540. doi: 10.3390/ijms160922527 26393575 10.3390/ijms160922527 PMC4613322

[12] Smolarczyk R, Cichon T, Jarosz M, Szala S. [HMGB1—its role in tumor progression and anticancer therapy]. Postepy Hig Med Dosw (Online). 2012; 66: 913–920. 23175347 10.5604/17322693.1021108

[13] Shin YJ, Kim MS, Kim MS, Lee J, Kang M, Jeong JH. High-mobility group box 2 (HMGB2) modulates radioresponse and is downregulated by p53 in colorectal cancer cell. Cancer Biol Ther. 2013; 14: 213–221. doi: 10.4161/cbt.23292 23255232 10.4161/cbt.23292 PMC3595303

[14] Costa-Silva B, Aiello NM, Ocean AJ, Singh S, Zhang H, Thakur BK, et al Pancreatic cancer exosomes initiate pre-metastatic niche formation in the liver. Nature Cell Biology. 2015; 17: 816–826. doi: 10.1038/ncb3169 25985394 10.1038/ncb3169 PMC5769922

[15] Gao J, Zou Z, Gao J, Zhang H, Lin Z, Zhang Y, et al Increased expression of HMGB3: a novel independent prognostic marker of worse outcome in patients with esophageal squamous cell carcinoma. Int J Clin Exp Pathol. 2015; 8: 345–352. 25755721 PMC4348853

[16] Gong Y, Cao Y, Song L, Zhou J, Wang C, Wu B. HMGB3 characterization in gastric cancer. Genet Mol Res. 2013; 12: 6032–6039. doi: 10.4238/2013.December.2.1 24338397 10.4238/2013.December.2.1

[17] Song N, Liu B, Wu JL, Zhang RF, Duan L, He WS, et al Prognostic value of HMGB3 expression in patients with non-small cell lung cancer. Tumour Biol. 2013; 34: 2599–2603. doi: 10.1007/s13277-013-0807-y 23609034 10.1007/s13277-013-0807-y

[18] Elgamal OA, Park JK, Gusev Y, Azevedo-Pouly AC, Jiang J, Roopra A, et al Tumor suppressive function of mir-205 in breast cancer is linked to HMGB3 regulation. PLoS One. 2013; 8: e76402 doi: 10.1371/journal.pone.0076402 24098490 10.1371/journal.pone.0076402 PMC3788717

[19] Yuan L, Zhou C, Lu Y, Hong M, Zhang Z, Zhang Z, et al IFN-gamma-mediated IRF1/miR-29b feedback loop suppresses colorectal cancer cell growth and metastasis by repressing IGF1. Cancer Lett. 2015; 359: 136–147. doi: 10.1016/j.canlet.2015.01.003 25592039 10.1016/j.canlet.2015.01.003

[20] Zhou C, Liu G, Wang L, Lu Y, Yuan L, Zheng L, et al MiR-339-5p regulates the growth, colony formation and metastasis of colorectal cancer cells by targeting PRL-1. PLoS One. 2013; 8: e63142 doi: 10.1371/journal.pone.0063142 23696794 10.1371/journal.pone.0063142 PMC3656035

[21] Zhang Z, Zhou C, Chang Y, Zhang Z, Hu Y, Zhang F, et al Long non-coding RNA CASC11 interacts with hnRNP-K and activates the WNT/beta-catenin pathway to promote growth and metastasis in colorectal cancer. Cancer Lett. 2016; 376: 62–73. doi: 10.1016/j.canlet.2016.03.022 27012187 10.1016/j.canlet.2016.03.022

[22] Chen X, Zhao G, Wang F, Gao F, Luo H, Wang Y, et al Upregulation of miR-513b inhibits cell proliferation, migration, and promotes apoptosis by targeting high mobility group-box 3 protein in gastric cancer. Tumour Biol. 2014; 35: 11081–11089. doi: 10.1007/s13277-014-2405-z 25095979 10.1007/s13277-014-2405-z

[23] Petit A, Ragu C, Della-Valle V, Mozziconacci MJ, Lafage-Pochitaloff M, Soler G, et al NUP98-HMGB3: a novel oncogenic fusion. Leukemia. 2010; 24: 654–658. doi: 10.1038/leu.2009.241 19956199 10.1038/leu.2009.241

